# Electrochemical and Ion Transport Studies of Li^+^ Ion-Conducting MC-Based Biopolymer Blend Electrolytes

**DOI:** 10.3390/ijms23169152

**Published:** 2022-08-15

**Authors:** Elham M. A. Dannoun, Shujahadeen B. Aziz, Mohamad A. Brza, Sameerah I. Al-Saeedi, Muaffaq M. Nofal, Kuldeep Mishra, Ranjdar M. Abdullah, Wrya O. Karim, Jihad M. Hadi

**Affiliations:** 1 Associate Chair of the Department of Mathematics and Science, Woman Campus, Prince Sultan University, P.O. Box 66833, Riyadh 11586, Saudi Arabia; 2 Hameed Majid Advanced Polymeric Materials Research Lab., Physics Department, College of Science, University of Sulaimani, Qlyasan Street, Kurdistan Regional Government, Sulaimani 46001, Iraq; 3 The Development Center for Research and Training (DCRT), University of Human Development, Sulaimani 46001, Iraq; 4 Medical Physics Department, College of Medicals & Applied Science, Charmo University, Chamchamal, Sulaimani 46023, Iraq; 5 Department of Chemistry, College of Science, Princess Nourah bint Abdulrahman University, P.O. Box 84428, Riyadh 11671, Saudi Arabia; 6 Department of Mathematics and Science, Prince Sultan University, P.O. Box 66833, Riyadh 11586, Saudi Arabia; 7 Department of Physics, Jaypee University, Anupshahar 203390, Uttar Pradesh, India; 8 Department of Chemistry, College of Science, University of Sulaimani, Qlyasan Street, Kurdistan Regional Government, Sulaimani 46001, Iraq; 9 Nursing Department, College of Nursing, University of Human Development, Kurdistan Regional Government, Sulaimani 46001, Iraq

**Keywords:** biopolymer blend electrolyte, EIS and FTIR, ion transport parameters, complex permittivity, LSV and TNM measurements

## Abstract

A facile methodology system for synthesizing solid polymer electrolytes (SPEs) based on methylcellulose, dextran, lithium perchlorate (as ionic sources), and glycerol (such as a plasticizer) (MC:Dex:LiClO_4_:Glycerol) has been implemented. Fourier transform infrared spectroscopy (FTIR) and two imperative electrochemical techniques, including linear sweep voltammetry (LSV) and electrical impedance spectroscopy (EIS), were performed on the films to analyze their structural and electrical properties. The FTIR spectra verify the interactions between the electrolyte components. Following this, a further calculation was performed to determine free ions (FI) and contact ion pairs (CIP) from the deconvolution of the peak associated with the anion. It is verified that the electrolyte containing the highest amount of glycerol plasticizer (MDLG3) has shown a maximum conductivity of 1.45 × 10^−3^ S cm^−1^. Moreover, for other transport parameters, the mobility (*μ*), number density (*n*), and diffusion coefficient (*D*) of ions were enhanced effectively. The transference number measurement (TNM) of electrons (*t*_el_) was 0.024 and 0.976 corresponding to ions (*t*_ion_). One of the prepared samples (MDLG3) had 3.0 V as the voltage stability of the electrolyte.

## 1. Introduction

Due to demand for high-energy consumption, for instance, to power laptops and mobile devices, the usage of energy storage devices is widespread. In order to produce low-cost and safe energy storage systems, the design of high-performance electrochemical devices has been extensively studied [1,2]. It is essential to use polymer electrolytes (PEs) for electrochemical devices because of their common advantages including qualities such as wide electrochemical windows, leakage-free, ability to form thin films, lightweight, flexibility, ease of handling, transparency, good conductivity, and solvent-free feature compared to commercial liquid electrolytes (LEs) [3,4]. In the PEs of the energy storage devices, the host polymer is often divided into two types: natural and synthetic polymers [5]. Non-biodegradable synthetic polymers deplete petroleum resources and introduce disposal difficulties [6]. As a result, biopolymers may be employed as the host polymer to investigate energy storage devices and minimize plastic waste pollution. These polymers, which derive from natural resources, have distinct advantages over synthetic ones, including low cost, wide compatibility with a wide range of solvents, abundance, and high film formation efficiency [7,8]. In PE investigations, starch, cellulose, chitosan, dextran, and carrageenan are the most- often employed biopolymers [9,10,11,12,13].

The search for novel ion-conducting PEs for lithium-based energy devices continues incessantly [14,15,16,17]. To replace the LEs in lithium-ion batteries, PEs that are linked to lithium salts and integrated into neutral or ion-conducting polymers have been suggested [18]. In contrast to manufactured polymers, which are durable, natural biopolymers degrade with time [19]. Cellulose is nature’s most abundant organic polymer, making it an excellent source of renewable energy [8]. As a natural polymer, cellulose is seen as a potential replacement for petrochemical polymers [20]. Cyanoacrylate is one of the most often used and lowest-priced types of cellulose. A biodegradable polymer that has excellent film-forming capabilities may be transparent and possesses superior mechanical and electrical properties that can be made from alkali cellulose. Methylcellulose (MC) is one of these cellulose derivatives [6]. By adding dimethyl sulfate or methyl chloride to alkali-based cellulose, a polymer with a 1,4 glycosidic link is created, known as MC [21]. Through a dative connection, ions create a complexation with polymer-host-oxygen-containing functional groups. Ion conduction in MC is facilitated by functional groups possessing lone-pair electrons, including hydroxyl, glycosidic link, and mexthoxy groups [22]. When it comes to film-forming and dissolving qualities, MC is a standout because of its strong mechanical, thermal, and chemical stabilities [23]. Glass transition temperature (Tg) for microcrystalline MC is between 184 and 200 °C, making it an excellent material for high-temperature applications [22]. Leuconostoc mesenteroides bacteria produce dextran, a non-toxic and biodegradable polysaccharide that has lone-pair electrons of heteroatoms, such as oxygen, which is essential for dissolving inorganic salts [13]. The polymer blend approach has been reported to generate a polymer mix host with higher ionic conduction sites [24]. A reduced glass transition temperature and degree of crystallinity may be achieved by mixing polymers [25]. A PE based on lithium salts is able to perform well overall in terms of crucial features, such as electrochemical window stability and ionic conductivity [26]. Various plasticizing agents were identified to further improve the above-mentioned properties. The loading of glycerol provided a conductivity of (1.32 ± 0.35) × 10^−3^ S cm^−1^ for the chitosan-PS-LiCF_3_SO_3_ system [27].

In this study, glycerol (contains three OH groups) as an eligible plasticizer has been used in an effort to pick up the conductivity of the blended polymer system. It causes weakening of the attraction force between the polymer chains and cations and anions of the salts [4]. The objective of this study is to enhance the conductivity of the prepared SPEs by adding glycerol as more ions are dissociated to increase conductivity. The electrochemical tests indicate the films are convenient for applications.

## 2. Results and Discussion

### 2.1. FTIR Results

**Figure 1:**
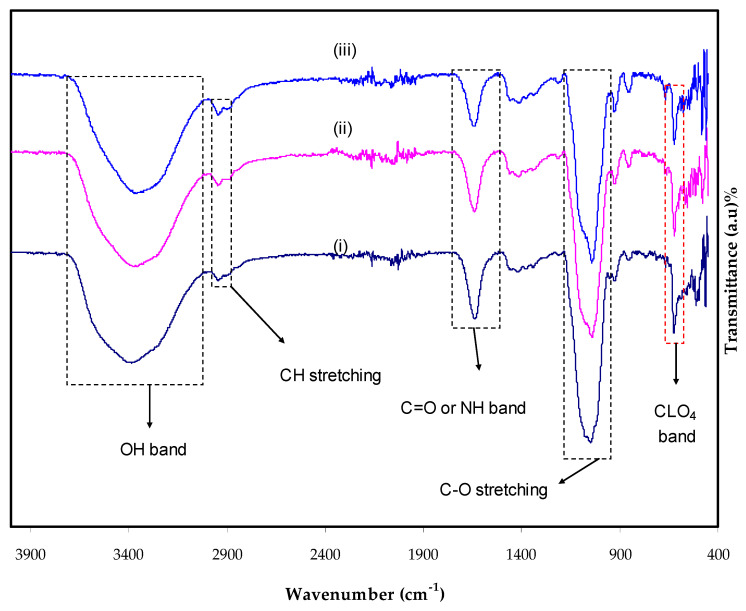
FTIR spectra for (i) MDLG1, (ii) MDLG2, and (iii) MDLG3 in the region 400–4000 cm^−1^.

To study polymer-mix developments, several scientists have turned to FTIR. Intermolecular interactions may be studied using FTIR spectroscopy, which analyzes spectra based on the stretching or bending vibrations of specific bonds. Figure 1 showed the spectra of the electrolytes at the 400 to 4000 cm^−1^. A wide band of 3353 cm^−1^ was observed in the FTIR spectra for MC: Dext, indicating the presence of OH groups [28,29]. The bands, due to -OH bending and -OH stretching, can be found at 1253–1503 cm^−1^ in a sharp peak and 3703–3149 cm^−1^ in a broad-peak form, respectively, by glycerol loading [30,31]. A peak at 1334 cm^−1^ came from -OH bending. The -CH asymmetrical and -CH symmetrical stretching are found at 3049 to 2849 cm^−1^ [32,33]. As the concentration of glycerol rises, the intensity of the -CH bands increases, indicating a complicated interplay between the glycerol and MC-Dex-LiClO_4_ [34]. As glycerol concentrations increase, the position of the electrolyte carboxamide and amine band shifts somewhat to 1749–1519 cm^−1^. The impact of increasing the content of glycerol on the strength of the interaction between the components of the polymer blend is proved where additional ions are interacting with the oxygen atoms and nitrogen atoms [27]. The range of carboxamide and amine bands can be recognized straightforwardly as reported by Aziz et al. [35] and Shukur et al. [36]. Interestingly, a sharp peak lies between 901 and 1203 cm^−1^ that comes from the C-O stretching, which is in accordance with the findings of the study documented by Mejenom et al. [37]. This band peak widens as the glycerol concentration increases. The insertion of LiClO_4_ salt into MC: Dex resulted in a significant shift in the strength of the bands, which is fascinating. The changes in the macromolecular arrangement have a direct effect on the intensity of these bands. The spectra of the complexes may show more and less organized structures, which might be the cause of these bands [38].

Many useful qualities, such as peak resolution, noise removal, and checking for interconnections between deconvolution parameters, are provided by the FTIR deconvolution, which is used in support of the conductivity findings [39]. When using this method, the deconvolution FTIR spectra may be used to determine the ion fraction that conducts electricity. Ramelli et al. noted that FTIR spectra might be deconvoluted, allowing one to isolate existing peaks and modify both intensity and wavenumber [40].

A peak for ClO_4_ localizes from 650 to 600 cm^−1^ and is regularly utilized in the investigation of ion–ion interactions in the PE and LiClO_4_ salt addition [41,42]. The ClO_4_ bands are featured by two peaks extending from 610 to 630 cm^−1^, which indicates that, at most, two dissimilar sorts of ClO_4_ anions are present in this material.

**Figure 2:**
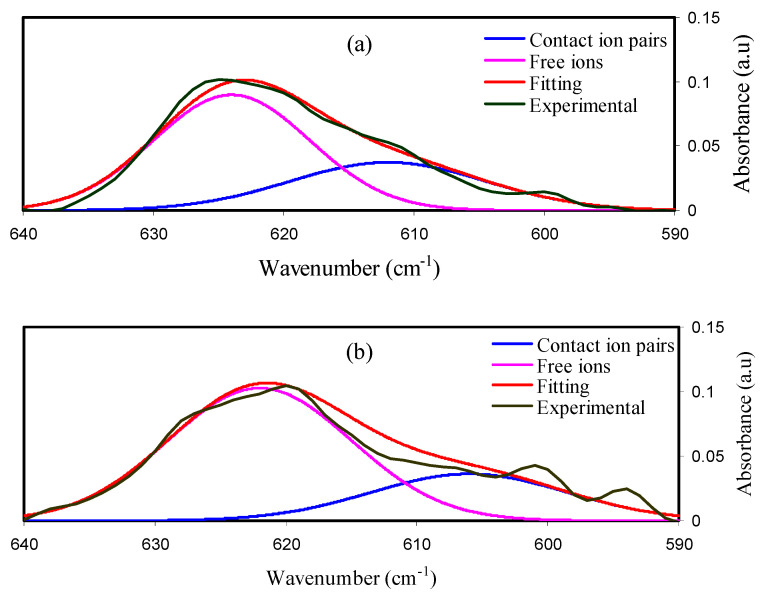

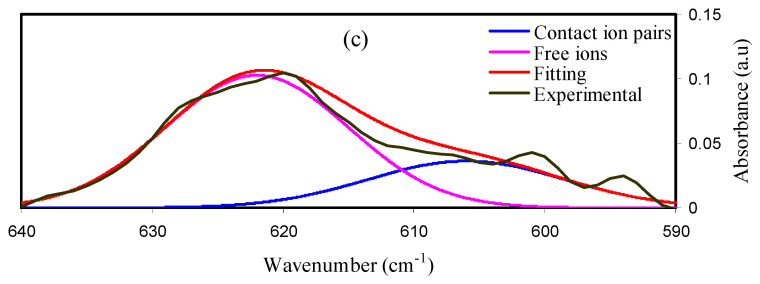
Deconvoluted FTIR spectra for (**a**) MDLG1, (**b**) MDLG2, and (**c**) MDLG3.

Salomon et al. documented that the presence of Li^+1^ is attached to the ClO_4_ band located at 610–630 cm^−1^. CIP ClO_4_^−1^ anions were observed at lower than 610 cm^−1^, while free ClO_4_ anions were observed at about 610–630 cm^−1^ [42]. Figure 2a–c show the deconvoluted FTIR spectra for the prepared electrolytes. The free ClO_4_ peaks are larger than the peaks of contact-ion pairs, as shown in Figure 2. Glycerol plasticizer helps dissolve LiClO_4_ salt in the MC: Dex matrix; therefore, this is what happens when the two mixtures are combined.

The free ions and contact ion pairs were measured using the area of the FTIR bands by the equations below [4]:

(1) $$\mathrm{Percentage}\;\mathrm{of}\;\mathrm{FI}\;(\%)\;=\frac{A_{f}}{A_{f}+A_{c}}\times 100\%$$

(2) $$\mathrm{Percentage}\;\mathrm{of}\;\mathrm{CIP}\;(\%)=\frac{A_{c}}{A_{f}+A_{c}}\times 100\%$$

where *A_f_* is the area of the FIP and *A_c_* is the area of the CIP. The percentages of FI and CIP are shown in Table 1.

The rise in ionic conductivity might also be attributed to the rise in Li^+^ ions that dissociate from LiClO_4_ salts. There is a strong correlation between the conductivity and the proportion of free ions, according to Aniskari and colleagues [43]. The calculation of the number density (*n*), ionic mobility (*µ*), and diffusion coefficient (*D*) for each electrolyte can be calculated from Equations (3)–(5). In these equations, *M* stands for the molecular weight of glycerol and *e* is the electron charge, and *N_A_* is the Avogadro’s constant. A polymer electrolyte has a total volume of *V_Total_*. The calculated values of *n*, *µ*, and *D* are shown in Table 2.

(3) $$n=\frac{M\times N_{A}}{V_{Total}}\times (freeion\%)$$

(4) $$\mu =\frac{\sigma }{ne}$$

(5) $$D=\frac{\mu kT}{e}$$

In Table 2 the *D*, *μ* and *n* values increase as the glycerol increases. The improvement of *D* and *μ* can be interpreted according to the increase in polymer chain flexibility upon the addition of the glycerol [1].

The relationship between the ionic conductivity of the electrolyte films and the ionic mobility is well-acknowledged and mathematically stated as follows:

(6) σ=∑ηqμ

where *σ* denotes the ionic conductivity, *η* represents the charge carrier density, and q stands for the single charge. From the equation above, it can be observed that the ionic conductivity improves with the increment of the ionic mobility as well as charge carrier density.

### 2.2. Impedance Study

**Figure 3:**
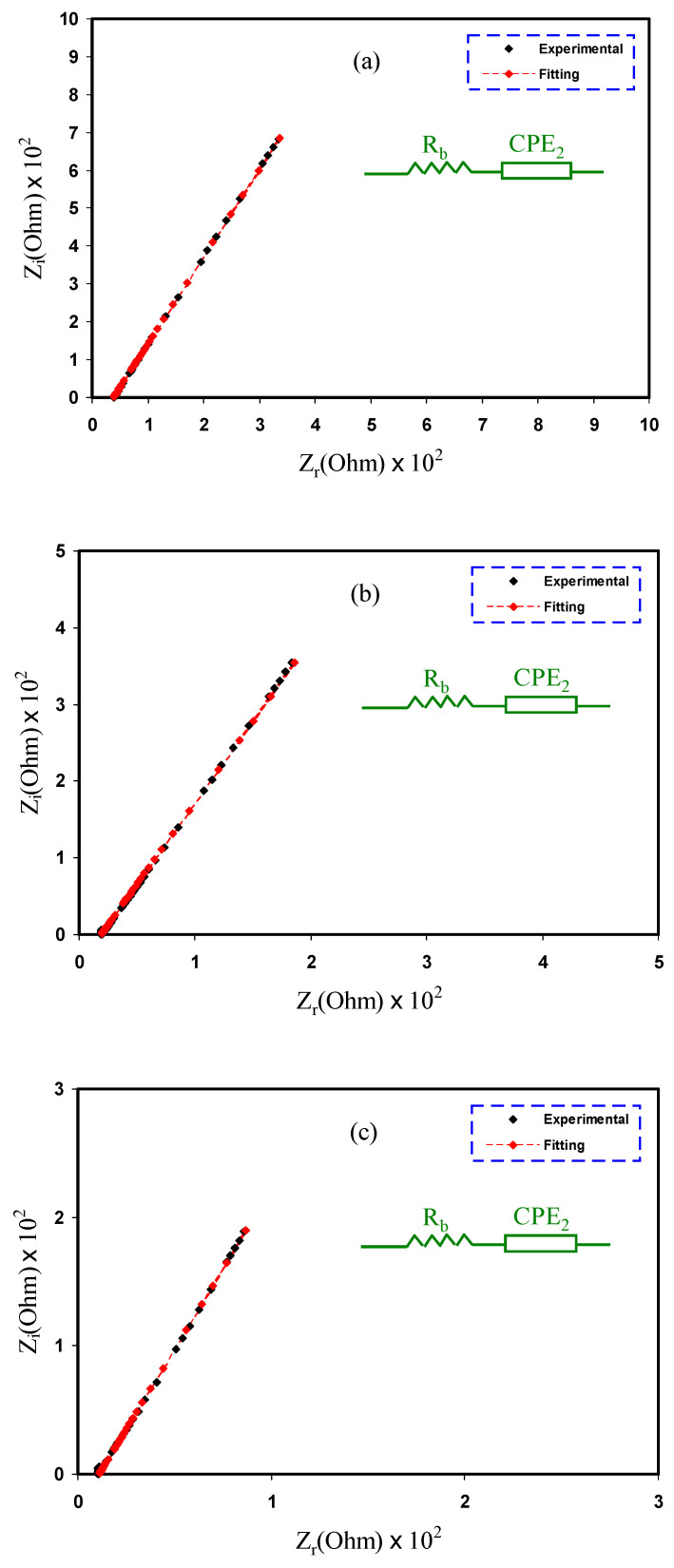
EIS spectra of (**a**) MDLG1, (**b**) MDLG2, and (**c**) MDLG3 electrolytes.

Figure 3 shows the Cole-Cole plots used to estimate the impedance parameters of the electrolytes used in this study. An appropriate equivalent circuit model, with series connections for the resistor and the capacitor given by bulk resistance (*R_b_*) and the constant phase element (*CPE*), is shown in the inset figure for each Cole-Cole plot. Ions flow via a resistor, whereas polymer chains remain immobile in a capacitor [44]. Because of the charge buildup and capacitive elements in the electrolytes, there is a spike in the plots created by the diffusion process inside the system [17,45]. This graph also shows how polarization and blocking electrodes in the Pes affect the inclination [46,47].

The impedance of *CPE* (*Z_CPE_*) is written as follows [48,49]:

(7) $$Z_{CPE}=\frac{1}{C\omega^{p}}\left[\cos\left(\frac{\pi p}{2}\right)-i\sin\left(\frac{\pi p}{2}\right)\right]$$

where *ω* denotes the angular frequency, *p* indicates the deviation of the plot from the axis, and *C* refers to the capacitance of *CPE* component. The spectra that involve only a spike and *R_b_* are in series with *CPE*, and the real and the imaginary parts of impedance, *Z_r_* and *Z_i_*, are based on the following mathematical relationships.

(8) $$Z_{r}=R+\frac{\cos\left(\frac{\pi p_{2}}{2}\right)}{C_{2}\omega^{p2}}$$

(9) $$Z_{i}=\frac{\sin\left(\frac{\pi p_{2}}{2}\right)}{C_{2}\omega^{p2}}$$

The determined *R_b_* and *CPE* for each electrolyte are listed in Table 3. The *CPE* values increase while the *R_b_* values fall when glycerol concentrations increased. There are more ions in a solution, resulting in a higher capacitance value, which results in the greater mobility and dissociation of ions, thereby increasing conductivity [50,51]. The ionic conductivity (*σ*) measured using Equation (10) and also shown in Table 3 demonstrates this.

(10) $$\sigma_{dc}=\left(\frac{1}{R_{b}}\right)\times \left(\frac{t}{A}\right)$$

Here, *t* refers to the electrolyte’s thickness; *A* denotes the SS electrodes area.

This study demonstrated that the MC:Dex:LiClO_4_:glycerol combination is more flexible and mobile because of the glycerol [52]. The conductivity of 1.99 × 10^−3^ S cm^−1^, achieved by MDLG3, is close to that achieved by Amran et al. [27] and Shukur et al. [30], and they also used glycerol as a plasticizer in their studies. It is also comparable with our previous studies of the biodegradable-blend-polymer electrolytes incorporated with ammonium salts [53,54]. The conductivity that was achieved in this study bodes well for future applications in energy devices [31].

As the samples have only a spike, *D*, *μ*, and *n* are measured by below equations [2]:

*D* is measured using Equations (11) and (12) [1]:

(11) $$D=D_{o}\exp\{-0.0297{\left[\ln D_{o}\right]}^{2}-1.4348\ln D_{o}-14.504\}$$

where the following is the case.

(12) $$D_{o}=\left(\frac{4k^{2}l^{2}}{R_{b}{}^{4}\omega_{\min}{}^{3}}\right)$$

Here, *ω*_min_ and l correspond to the angular frequency that is based on the minimum *Z_i_* and the electrolyte thickness, respectively. *µ* is measurable from the relationship shown in Equation (13):

(13) $$\mu =\left(\frac{eD}{K_{b}T}\right)$$

where *K_b_* and *T* are the Boltzmann constant possess normal meanings.

The conductivity can be measured using Equation (6).

Thus, the number *n* is measured using Equation (14):

(14) $$n=\left(\frac{\sigma_{dc}K_{b}T\tau_{2}}{{\left(eK_{2}\varepsilon_{o}\varepsilon_{r}A\right)}^{2}}\right)$$

In Table 4, *D*, *μ*, and *n* increased when glycerol increased. This is caused by increasing the polymer chain’s flexibility when the glycerol is loaded [2]. The outcome shows how the concentration of glycerol affects the values of the ion number density, the ionic mobility, and the diffusion coefficient. This increase in *D*, *μ*, and *n* values can cause an increase in conductivity [4]. It is interesting to observe that when glycerol concentration increases, the number of ions (*n*) tends to increase continuously. Glycerol enhances the dissociation of salts to free ions; thus, *n* increases correspondingly. Meanwhile, ionic mobility (*μ*) and diffusion coefficient (D) are observed to follow the same trend of ionic conductivity, as shown in Table 3. The value of the free ion, which gradually increased by adding glycerol to the system, indicates that the ionic conductivity of the present system increased by the increasing the (*n*) value. These results from EIS and the FTIR deconvolution are in agreement.

### 2.3. Dielectric Properties

**Figure 4:**
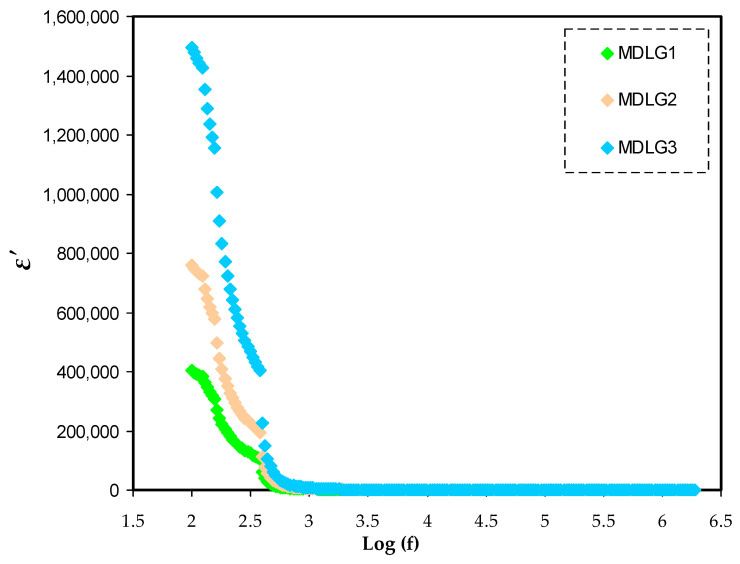
*ε*′ spectra versus frequency for MC:Dex:LiClO_4_:Glycerol electrolytes.

**Figure 5:**
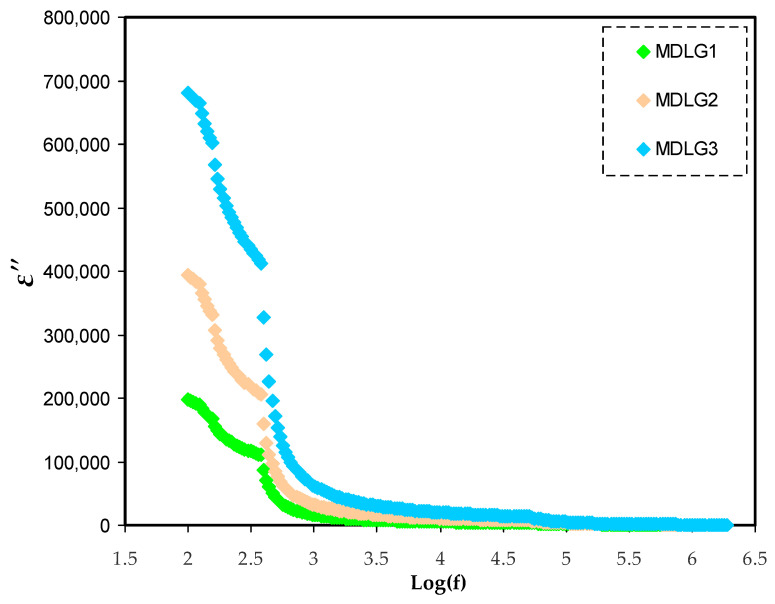
*ε*″ spectra versus frequency for MC:Dex:LiClO_4_:Glycerol electrolytes.

According to current research, dielectric material qualities may be defined in multiple ways. There were many ways to increase the accuracy and sensitivity of material characterization [55,56,57,58,59]. Impedance studies at various frequencies have been shown to be a good approach for studying the molecular mobility of dielectric materials [60]. Dielectric studies may be used to examine the conductivity trend. Different amounts of glycerol at ambient temperature affect the dielectric constant (*ε*′) and the dielectric loss (*ε*″), as observed in Figure 4 and Figure 5, respectively. *ε*′ and *ε*″ are measured using the equations below [61,62,63]:

(15) $$\varepsilon^{\prime }=\left[\frac{Z^{\prime \prime }}{\omega C_{o}({Z^{\prime }}^{2}+{Z^{\prime \prime }}^{2})}\right]$$

(16) $$\varepsilon^{\prime \prime }=\left[\frac{Z^{\prime }}{\omega C_{o}({Z^{\prime }}^{2}+{Z^{\prime \prime }}^{2})}\right]$$

where *C*_o_ is the vacuum capacitance, which is equivalent to $\varepsilon_{0}A/t$ in which $\varepsilon_{0}$ is the vacuum permittivity; the angular frequency is denoted by *ω* (*ω* = 2πf); the frequency is denoted by f.

The conductivity of a polymer electrolyte is determined by its dielectric constant [64]. Real dielectric permittivity (*ε*′) is used to determine the polarization or dipole alignment, which is measured by capacitance. Similarly to *ε*″, which indicates dielectric loss, conductance reflects the energy needed to align dipoles in a dielectric medium [65]. An important consideration in electrical conductivity testing is the identification of neutral ion pairs produced by the interaction of dissolved ion pairs [59]. In EIS measurements, it was shown that by adding more glycerol, the DC’s conductivity significantly increased. *ε*′ and *ε*″ at low frequencies are higher (Figure 4 and Figure 5), which show variations for the films. Charge carriers or space charge polarization build up at the electrode/electrolyte contact point, causes this phenomenon [60]. Increasing the frequency reduces the dielectric property (bulk property). As a result, *ε*′ and *ε*″ increase as a result of a decrease in the frequency of the applied electric field [66]. As a result of the quick reversal of the electric field frequency, there is no new ion diffusion that takes place along its route, and polarization is reduced. Eventually, the peak shrinks to the point where it is no longer frequency dependent [48]. In a comparison to other samples, the system containing 42 wt.% of glycerol had a greater dielectric constant. Dielectric loss (*ε*″) and constant (*ε*′) are strongly impacted by the conductivity in the system [51,67].

It has previously been observed that the dielectric constant (*ε*′) and the density of the charge carriers (*n_i_*) were formulated by the following relationship:

(17) $$n_{i}=n_{o}\exp(-U/\varepsilon^{\prime }K_{b}T)$$

where *U* is the dissociation energy.

DC’s conductivity, as well as dielectric constant values, can be manipulated successfully [68]. The dielectric constants of polymer electrolytes may be used to determine the conductivity of certain materials and, hence, their electrical properties. A drop in dielectric constant is accompanied with a decrease in capacitance (*ε*′ = *C*/*C*_o_). The plots show that the *ε*″ value is higher than the *ε*′, as shown in Figure 4 and Figure 5. DC conduction processes and dielectric polarization processes both have an impact on dielectric loss [51].

### 2.4. TNM Study

**Figure 6:**
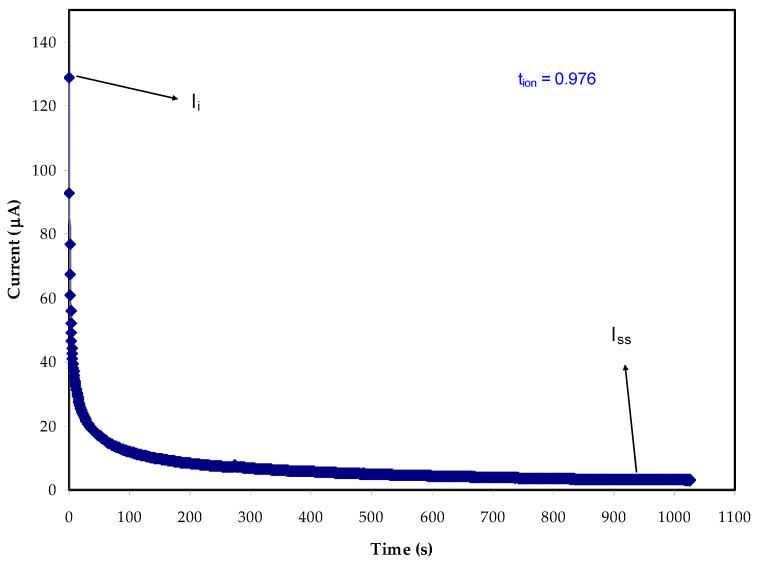
Chronoamperometric profile of for the MDLG3 electrolyte.

In order to ensure the purely ionic nature of the PE system, the ion transport number (*t_ion_*) has been measured for the optimized MC:Dex: LiClO_4_:Glycerol composition using the DC polarization technique [69]. The curve obtained for the SS|Polymer electrolyte|SS cell (SS: stainless-steel) is shown in Figure 6. An initial current (*I_i_*) of 128 A and the total of ionic and electronic currents were delivered by the cell. Since the SS electrode is ion-blocking in nature, the current declines quickly and is saturated at the residual electronic current (*I*_e_) of 3 µA. The electrolyte system’s ionic composition is thought to be responsible for the abrupt reduction in current levels. The *t_ion_* and *t_el_* values of the electrolyte film, obtained using the Equations (18) and (19), are found to be 0.976 and 0.024, respectively. These results show how ionic the electrolyte system is and how it will protect the electrodes of the energy storage device from each other since it is close to 1, which means it is close to the ideal value of unity. Ions are the key charge carriers in this system of methylcellulose-dextran-LiClO_4_:Glycerol [70,71,72]. The result obtained in this study is observed to be high compared to our previous study for methylcellulose-based polymer electrolytes impregnated with potassium iodide [73].

Equations (17) and (18) are used to measure *t_ion_* and *t_el_*.

(18) $$t_{ion}=\frac{I_{i}-I_{ss}}{I_{i}}$$

(19) $$t_{el}=1-t_{ion}$$

In Equations (18) and (19), the starting and the steady-state current are expressed as *I_i_* and *I_ss_*, respectively.

**Figure 7:**
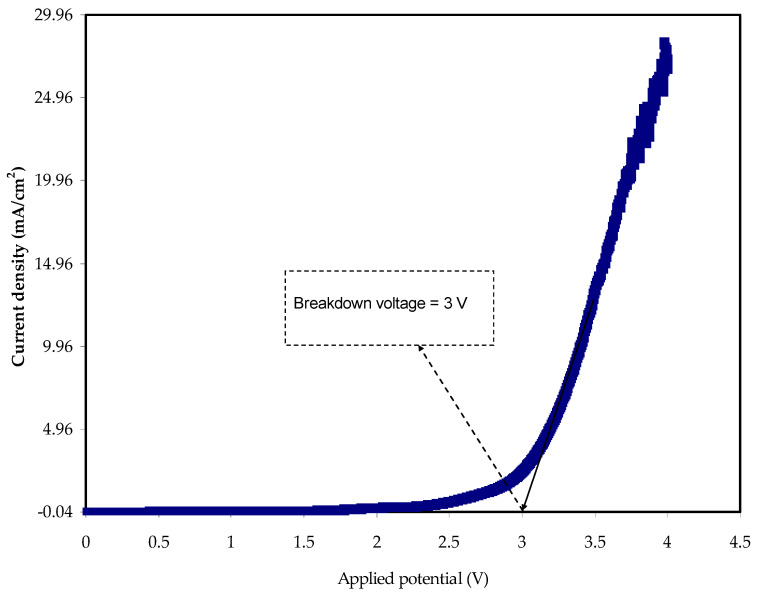
LSV for the MDLG3 film of SPE.

The electrochemical stability window (ESW) is an important parameter for an electrolyte, which determines the working voltage range of the energy storage device. The ESW of the optimized MC:Dextran:40 wt.% LiClO_4_:48 wt.% Glycerol composition is obtained using linear sweep voltammetry (LSV). The LSV curve, shown in Figure 7, displays a plateau of negligible current without any anodic/cathodic current peak up to ~3 V. The present values increase sharply after the aforementioned potential. A considerable ESW of 3 V is shown, making the electrolyte film acceptable for supercapacitor use. In order for the film to be used in energy storage devices, the stability of the plasticized methylcellulose-dextran-LiClO_4_ system has been shown to be up to 3 V. The interesting observation in this study is the eligibility of the MDLG3 electrolyte for energy storage device utilization. This is caused by the satisfactory voltage breakdown of the sample at almost 1.0 V [74,75]. The decomposition voltage attained in this study is relatively high compared to our previous studies [76,77]. This could be due to the presence of LiClO_4_ as an ionic source, which has higher stability than ammonium salts. Moreover, a protic ionic liquid electrolyte was utilized for lithium-ion batteries as documented by Bockenfeld et al. [78]. They demonstrated that the highest potential stability was 2.65 V for their electrolyte that incorporated 0.5 M lithium nitrate (LiNO_3_) in propylene carbonate-pyrrolidinium nitrate.

## 3. Materials and Methods

### 3.1. Materials

MC polymer (*M*_w avg_ = 10,000–220,000), LiClO_4_ (*M*_w_ = 106.39 g/mol) and glycerol (*M*_w_ = 92.09382 g/mol) were purchased from Sigma-Aldrich (Kuala Lumpur, Malaysia).

### 3.2. Electrolyte Preparation

The synthesis of MC-Dex-blend polymer was performed by stirring and dissolving 40 wt.% of Dex (0.4 g) and 60 wt.% of MC (0.6 g) individually, each in a 1% solution of 30 mL acetic acid, for almost 2 h at room temperature. Then, the two solutions were stirred and blended using a magnetic stirrer for around 4 h until reaching a homogenous-blend solution. Then, with respect to the above solution, 40 wt.% (0.666 g) of LiClO_4_, MC-Dex-LiClO_4_ formed. Ultimately, in the step of 14 wt.%, 14, 28, and 42 wt.% of glycerol were added to the MC-Dex-LiClO_4_ solution followed by continuous stirring until the synthesis of plasticized SPEs was achieved. The labelling of the series of the samples was conducted as follows: MDLG1, MDLG2, and MDLG3 for the MC-Dex-LiClO_4_ loading 14, 28, and 42 wt.% of glycerol, respectively as shown in Table 5. The casting of the series of sample solutions was carried out in the Petri dishes, followed by leaving them at room temperature to evaporate the solvent gradually. The free solvent sample films were kept in a desiccator.

### 3.3. Methods of Characterizations

#### 3.3.1. FTIR and EIS Measurements

The FTIR spectra of the blended polymer systems were acquired using FTIR Spectrophotometer (Malvern Panalytical Ltd., Malvern, UK), ranging from 4000 to 400 cm^−1^ with a resolution of 2 cm^−1^. The EIS samples spectra were acquired using the EIS (3532-50 LCR HiTESTER (HIOKI), Nagano, Japan) within 50 Hz and 5,000,000 Hz of frequency. The circle film had a geometric circle shape (diameter of 2 cm), which was sandwiched between stainless steel (SS) electrodes using a spring force during electrochemical measurements. The cell was hyphenated with a computer to measure real and imaginary (Z′ and Z″) parts of the complex impedance spectra (Z*).

#### 3.3.2. TNM and LSV

The ion (*t_ion_*) and electron (*t_el_*) transference numbers were measured precisely. The cell (SS|MDLG3|SS) was connected to the UNI-T UT803 multimeter and A&V Instrument DP3003 digital DC power supply. By applying a voltage of 0.2 V to the cell, the polarization of the cell was obtained over a sufficient amount of time at room temperature. To obtain the potential stability of the MDLG3, LSV was used by applying 10 mV s^−1^ within 0.0 and 4.0 V. The cell was the three-electrode type, and the working, counter, and reference electrodes were used by utilizing the Digi-IVY DY2300 potentiostat. The current changes over the mentioned potential were obtained.

## 4. Conclusions

In this study, SPEs based on MC:Dex:LiClO_4_ plasticized with glycerol were synthesized by the solution-cast method. The conductivity increased to 1.45 × 10^−3^ S cm^−1^ due to the doping of glycerol. The FTIR method showed that there was an interaction of LiClO_4_ and glycerol with the MC and Dex by changing FTIR absorption peaks. The FTIR deconvolution of CLO_4_^−^ anions showed that the free ion percentages increased when glycerol increased, while the percentages of contact ion pairs decreased. Further proof of *DC* conductivity trends was emphasized from the dielectric measurement. The addition of glycerol was effective in increasing the number density (*n*), diffusion coefficient (*D*), and mobility (*μ*). Additionally, the mass transport improvements of the electrolytes originate from the increase in chain flexibility. The values of measured *t_ion_* and *t_el_* indicate the ion’s responsibility for conduction in the polymer-electrolyte system. The stability voltage range of the electrolyte system is satisfactory, meaning that the SPE is eligible for utilization at large scales in electrochemical energy storage devices.

## Figures and Tables

**Table 1:** The percentages of ions.

Sample	FI%	CIP%
MDLG1	65.85%	34.14%
MDLG2	72.58%	27.42%
MDLG3	76.95%	23.05%

**Table 2:** The *n*, *D*, and *μ* at ambient temperature from FTIR approach.

Glycerol %	*n* (cm^−3^)	*µ* (cm^2^ V^−1^ s)	*D* (cm^2^ s^−1^)
MDLG1	2.32 × 10^22^	4.0 × 10^−8^	1.04 × 10^−9^
MDLG2	5.92 × 10^22^	1.09 × 10^−7^	2.83 × 10^−9^
MDLG3	1.13 × 10^23^	1.10 × 10^−7^	2.86 × 10^−9^

**Table 3:** EEC fitting parameters for each sample.

Sample	K (F^−1^)	*CPE* (F)	*R_b_* (Ohm)	Conductivity (S cm^−1^)
MDLG1	5.21 × 10^4^	1.92 × 10^−5^	3.80 × 10^1^	3.93 × 10^−4^
MDLG2	2.45 × 10^4^	4.08 × 10^−5^	1.89 × 10^1^	8.16 × 10^−4^
MDLG3	1.59 × 10^4^	6.29 × 10^−5^	1.10 × 10^1^	1.45 × 10^−3^

**Table 4:** The values of ion transport parameters of each film from impedance approach.

Sample	*D* (cm^2^ s^−1^)	*µ* (cm^2^ V^−1^ s)	*n* (cm^−3^)
MDLG1	1.72 × 10^−7^	6.71 × 10^−6^	3.65 × 10^20^
MDLG2	1.88 × 10^−7^	7.33 × 10^−6^	6.95 × 10^20^
MDLG3	2.64 × 10^−7^	1.03 × 10^−5^	8.80 × 10^20^

**Table 5:** The identification and composition for the MC-Dex-LiClO_4_–glycerol systems.

Sample Code	MC (g)	Dex (g)	LiClO_4_ (g)	Glycerol (g)	Glycerol wt.%
MDLG1	0.6	0.4	0.666	0.271	14
MDLG2	0.6	0.4	0.666	0.647	28
MDLG3	0.6	0.4	0.666	1.206	42

## Data Availability

Not applicable.
